# Geographical Differences in the Epidemiology and Treatment of Candida Prosthetic Joint Infections

**DOI:** 10.1093/ofid/ofag208

**Published:** 2026-04-15

**Authors:** Christel Mamona-Kilu, Jaime Lora-Tamayo, Martin McNally, Clara Duran, Rosemary Ho, Matthew Scarborough, Maria Dudareva, Eric Bonnet, Laura Escolà-Vergé, Dolores Rodriguez Pardo, Gerald Jesuthasan, Eric Senneville, Pauline Thill, Stéphane Klein, Cécile Ronde-Oustau, Maria Luisa Sorlí Redó, Stéphane Corvec, Nicolò Rossi, Jaime Esteban, Adrien Lemaignen, Taiana Cunha Ribeiro, Julien Mazet, Milène Sasso, Mauro José Costa Salles, Mikel Mancheño-Losa, Gérard Giordano, Bernhard J H Frank, Jochen G Hofstaetter, José María Barbero Allende, Olivier Lortholary, Camille Fourcade, Laura Morata, Alex Soriano, Aurélien Dinh, European Society of Clinical Microbiology and Infectious Diseases Study Group on Implant Associated Infections (ESGIAI), Thomas Bauer, Camille Courboulès, Emma d’Anglejan, Aurélien Dinh, Clara Duran, Christel Mamona Kilu, Latifa Noussair, Anne-Laure Roux, Eric Bonnet, Camille Fourcade, Gérard Giordano, Maria Dudareva, Rosemary Ho, Gerald Jesuthasan, Martin McNally, Matthew Scarborough, Bernhard J H Frank, Jochen G Hofstaetter, Stephane Klein, Cecile Ronde Oustau, Éric Senneville, Pauline Thill, Laura Escolà-Vergé, Dolores Rodriguez Pardo, Laura Morata, Alex Soriano, Etienne Canouï, André Paugam, Gertrude Touanga, Pierre Delobel, Jaime Lora-Tamayo, Mikel Mancheño-Losa, Jean-Philippe Lavigne, Milène Sasso, Julien Mazet, Albert Sotto, Juan Gomez Junyent, Maria Luisa Sorlí Redó, Mauro José Costa Salles, Taiana Cunha Ribeiro, José Maria Barbero Allende, Guillaume Desoubeaux, Adrien Lemaignen, Chloé Porche, Cédric Arvieux, Anne Méheut, Jean-Pierre Gangneux, Carine Couzigou, Julie Lourtet, Benoît Pilmis, Justinas Stucinskas, Danguole Vaznaisiene, Nicolò Rossi, Stéphane Corvec, Vincent Crenn, Florent Morio, Marta Fernández-Sampedro

**Affiliations:** Infectious Disease Department, Raymond-Poincaré University Hospital, Paris Saclay University, Assistance Publique-Hôpitaux de Paris (AP-HP), Garches, France; Infectious Disease Department, Infectious Disease Hospital Universitario 12 de Octubre, Madrid, Spain; Nuffield Orthopaedic Centre, Oxford University Hospitals, Oxford, UK; Infectious Disease Department, Raymond-Poincaré University Hospital, Paris Saclay University, Assistance Publique-Hôpitaux de Paris (AP-HP), Garches, France; Nuffield Orthopaedic Centre, Oxford University Hospitals, Oxford, UK; Nuffield Orthopaedic Centre, Oxford University Hospitals, Oxford, UK; Nuffield Orthopaedic Centre, Oxford University Hospitals, Oxford, UK; Infectious Disease, Joseph Ducuing Hospital, Toulouse, France; Infectious Diseases Department, Hospital de la Santa Creu i Sant Pau, Barcelona, Spain; Infectious Diseases Department, Hospital de la Santa Creu i Sant Pau, Barcelona, Spain; Nuffield Orthopaedic Centre, Oxford University Hospitals, Oxford, UK; Infection Disease, University Hospital, Tourcoing, France; Infection Disease, Lille University Hospital, Lille, France; Infectious Disease, Strasbourg University Hospital, Strasbourg, France; Infectious Disease, Strasbourg University Hospital, Strasbourg, France; Infectious Disease, Hospital del Mar, Barcelona, Spain; Microbiology Department, Nantes University Hospital, Nantes, France; Infectious Diseases Unit, Guglielmo da Saliceto Hospital, Piacenza, Italy; Clinical Microbiology, Hospital Universitario Fundación Jiménez Díaz, Madrid, Spain; Infectious Disease Department, Bretonneau University Hospital, Tours, France; Infectious Disease, Faculdade de Ciências Médicas Santa Casa de São Paulo, São Paulo, Brasil; Infectiopus Disease, Caremeau University Hospital, Nîmes, France; Infectiopus Disease, Caremeau University Hospital, Nîmes, France; Infectious Disease, Faculdade de Ciências Médicas Santa Casa de São Paulo, São Paulo, Brasil; Infectious Disease Department, Infectious Disease Hospital Universitario 12 de Octubre, Madrid, Spain; Orthopedic Surgery, Joseph Ducuing Hospital, Toulouse, France; Laboratory for Orthopaedic Research, Orthopaedic Hospital Vienna, Speising, Austria; Laboratory for Orthopaedic Research, Orthopaedic Hospital Vienna, Speising, Austria; Infectious Disease, Príncipe de Asturias University Hospital, Madrid, Spain; Necker-Pasteur Center for Infectious Diseases and Tropical Medicine, Necker-Enfants Malades University Hospital, AP-HP, Paris, France; Institut Pasteur, Centre National de Référence Mycoses, Paris, France; Groupe de Recherche Translationnelle en Mycologie, Mycology Department, Invasives et Antifongiques, Paris Cité University, Paris, France; Infectious Disease, Joseph Ducuing Hospital, Toulouse, France; Hospital Clínic de Barcelona, Barcelona, Spain; Hospital Clínic de Barcelona, Barcelona, Spain; Infectious Disease Department, Raymond-Poincaré University Hospital, Paris Saclay University, Assistance Publique-Hôpitaux de Paris (AP-HP), Garches, France

**Keywords:** candida, epidemiology, fluconazole, prosthetic joint infection, surgery

## Abstract

**Background:**

Management of prosthetic joint infections (PJIs) due to *Candida* spp. remains challenging and poorly standardized. Epidemiological patterns and therapeutic strategies may vary between centers and countries, potentially reflecting differences in access to antifungal agents.

**Methods:**

We performed a secondary analysis of an international, multicenter, retrospective study supported by the European Society of Clinical Microbiology and Infectious Diseases, including *Candida* PJI diagnosed between 2010 and 2020. Cases met European Bone and Joint Infection Society criteria, combining clinical signs of infection with at least 2 intraoperative samples positive for *Candida* spp. Follow-up was 2 years. Epidemiology, management, and outcomes were compared across 5 groups: France, Spain, England, Austria, and other countries.

**Results:**

Overall, 268 cases were included: France (n = 142), Spain (n = 42), England (n = 38), Austria (n = 36), and others (Brazil, Lithuania, Italy; n = 9). Distribution of infected sites was similar across countries (hip 53.4%, knee 43.3%, and other 3.3%), as was species epidemiology (*Candida albicans* 55.6%, *Candida parapsilosis* 29.5%, *Candida glabrata* 7.8%, and *Candida tropicalis* 5.6%). Surgical strategies differed: 1-stage exchange was more frequent in France (36.0%) and Austria (34.3%), whereas 2-stage exchange predominated in England (42.1%) and Spain (37.2%). Echinocandins were prescribed significantly more often in France (41.8%) than elsewhere. Overall outcomes were poor, with a global failure rate of 43%, without significant differences between countries.

**Conclusions:**

International differences in epidemiology and management of *Candida* PJI appear limited. Variations in surgical and antifungal strategies did not translate into improved outcomes, highlighting the need for optimized and standardized management approaches in future collaborative prospective studies worldwide and clinical.

## BACKGROUND

Prosthetic joint infections (PJIs) due to *Candida* spp. are rare [1–4]. Epidemiological, microbiological, and management-related factors may vary according to countries and impact outcome. Few comparisons between different countries are available in literature [1, 2].

Regarding bloodstream infections due to *Candida*, there is considerable variation among species and antifungal treatment resistance [5]. Indeed, recent European surveillance data highlight substantial geographic heterogeneity in the epidemiology and antifungal susceptibility of candidemia. While *Candida albicans* remains the predominant species overall, its relative proportion continues to decline in many regions, paralleling a rise in the number of *Candida glabrata* cases in Northern and Central Europe and *Candida parapsilosis* cases particularly in Southern Europe [5]. These species shifts are clinically relevant given their distinct resistance profiles: *C glabrata* exhibits consistently elevated rates of acquired fluconazole resistance across multiple countries, whereas *C parapsilosis* demonstrates rapidly emerging fluconazole resistance with documented clonal dissemination in Greece, Italy, and Turkey [5]. Although echinocandin resistance remains uncommon, sporadic isolates harboring FKS hotspot mutations, including novel alterations in *C parapsilosis*, indicate a potential emergence of multidrug resistance, especially in settings of high echinocandin use [5]. Together, these findings underscore pronounced intercountry differences in species distribution and antifungal resistance, reinforcing the need for up-to-date locally informed surveillance to guide empirical therapy and antifungal stewardship efforts [5].

Thus, we performed a country-based analysis of the largest multicenter study available to date on PJI due to *Candida* species to describe and compare local epidemiology and treatment [1].

## METHODS

We conducted an international, multicenter, retrospective study supported by the European Society of Clinical Microbiology and Infectious Diseases (ESCMID), including cases of *Candida* PJI diagnosed between 2010 and 2020. The survey was disseminated through the mailing lists of 2 ESCMID groups (European Study Group for Implant Associated Infections and ESCMID Study Group for Infections in the Elderly), as well as through our professional networks. All respondents were included as long as they fulfilled the predefined inclusion criteria. PJI case definition followed European Bone and Joint Infection Society criteria: the presence of clinical signs of infection and at least 2 intraoperative specimens yielding *Candida* spp. [6, 7]. Immunosuppression was defined as the presence of asplenia, neutropenia, agammaglobulinemia, solid organ transplantation, hematological malignancy, known human immunodeficiency virus infection with a CD4+ count <400 cells/μL, or Child–Pugh class C cirrhosis.

Patients were followed for 2 years, and treatment failure was defined using a composite endpoint: recurrence of clinical signs of infection with *Candida* isolation, initiation of suppressive antifungal therapy, or death from infectious causes. Infective mortality was defined according to the investigator's clinical judgment at each participating center.

We subsequently conducted an analysis comparing epidemiology, management, and outcomes across 5 groups: France, Spain, England, Austria, and all remaining countries (Lithuania, Brazil, and Italy).

A standardized questionnaire collected demographic, clinical, and microbiological data, as well as therapeutic management and outcomes. It was distributed through ESCMID, with voluntary participation from centers.

Comprehensive methodological procedures and definitions are available in a previously published report [1].

### Statistics

Statistical analyses were conducted to compare demographic, clinical, microbiological, therapeutic, and outcome variables across participating countries. Continuous variables were presented as medians with interquartile ranges (IQRs) and compared using the Kruskal–Wallis test owing to their nonnormal distribution. Categorical variables were presented as frequencies and percentages and compared using Pearson's χ^2^ test; Fisher's exact test was applied when expected cell counts were <5. To assess geographical variation in *Candida* species distribution, a global χ^2^ test was performed across all species categories.

To evaluate whether intercountry differences in species distribution persisted after adjustment for potential confounders, a multinomial logistic regression model was constructed with *Candida* species as the dependent variable and country as the main independent variable. Model fit and multicollinearity were assessed, and robust standard errors were used when appropriate. All tests were 2-sided, and a *P*-value <.05 was considered statistically significant.

Statistical analyses were performed using standard software packages (eg, R software, version 4.3.2), and no imputation was applied for missing data given the descriptive nature of the study.

### Ethics

This study received approval from the French Infectious Disease Society Institutional Review Board (IRB00011642). Specific local permissions from the ethics review committee of the promoting center were obtained prior to recruitment start. Patient data were anonymized, and all information were handled in accordance with European and local data protection regulations, including General Data Protection Regulation and Commission Nationale de l’Informatique et des Libertés (Reference Method 004) in France.

## RESULTS

A total of 268 patients with *Candida* PJI were included across 7 countries. Several patient characteristics differed significantly by country (Table 1). Median age varied across settings (*P* = .025), with Austria (76 years) and Spain (74 years) having substantially older cohorts, whereas Brazil had younger patients (56 years). Sex distribution also differed significantly (*P* = .003): Austria showed a strikingly low proportion of male patients (19.4%), in contrast with the United Kingdom (57.9%) and Brazil (60.0%).

**Table 1.** Population Characteristics of Pji Due to Candida According to Country

Characteristics	Overall (N = 268)	Austria (N = 36)	Brazil (N = 5)	France (N = 142)	Italy (N = 1)	Lithuania (N = 3)	Spain (n = 43)	United Kingdom (N = 38)	*P-*Value
Age (median)	73 (64–79)	76 (71–79)	56 (49–72)	73 (62–79)	79	74 (74–78)	76 (68–82)	69 (63–75)	.025
Male	125 (46.6%)	7 (19.4%)	3 (60.0%)	71 (50.0%)	0	0	22 (51.2%)	22 (57.9%)	.003
Charlson score	4 (2–5)	4 (3–5)	3 (2–4)	3 (2–5)	7	4 (4–5)	4 (2–5)	4 (2–5)	.4
Diabetes mellitus, n (%)	72 (26.9)	7 (19.4)	2 (40.0)	35 (24.6)	0 (0.0)	1 (33.3)	12 (27.9)	15 (39.5)	.421
Immunosuppression	29 (10.8%)	1 (2.8%)	1 (20.0%)	13 (9.2%)	1	…	2 (4.7%)	11 (28.9%)	.001
Time between previous surgery and index infection (median)	59.0 (24.0–385.0)	82.5 (33.0–478.5)	126.0 (50.0–188.5)	44.0 (22.0–357.0)	NA (NA–NA)	53.0 (22.0–236.0)	149.0 (26.0–587.0)	63.0 (31.0–243.0)	.454
Localization									
Hip	143 (53.4)	21 (58.3%)	3 (60.0%)	76 (53.5%)	1	2 (66.7%)	27 (62.8%)	13 (34.2%)	.2
Knee	116 (43.3%)	15 (41.7%)	2 (40.0%)	60 (42.3%)	0	1 (33.3%)	15 (34.9%)	23 (60.5%)	.7
Shoulder	4 (1.5%)	0	0	2 (1.4%)	0	0	1 (2.3%)	1 (2.6%)	.3
Tibia	2 (0.7%)	0	0	2 (1.4%)	0	0	0	0	>.9
Femur	2 (0.7%)	0	0	1 (0.7%)	0	0	0	1 (2.6%)	.5
Foot	1 (0.4%)	0	0	1 (0.7%)	0	0	0	0	.8
Previous infection	203 (75.7%)	23 (63.9%)	1 (20.0%)	122 (85.9%)	0	2 (66.7%)	23 (53.5%)	32 (84.2%)	< .001
Previous infection due to Candida	13 (6.3%)	7 (30.4%)	NA	2 (1.6%)	NA	NA	0	4 (12.5%)	< .001
Candida species									
*albicans*	149 (55.6%)	15 (41.6%)	4 (80.0%)	84 (59.2%)	1	3 (100%)	21 (48.8%)	21 (50.3%)	0.2
*glabrata*	21 (7.8%)	3 (8.3%)	0	10 (7.0)	0	0	3 (7.0%)	5 (13.2%)	.8
* parapsilosis*	79 (29.5%)	16 (44.4%)	0	37 (26.1)	0	0	17 (39.5%)	9 (23.7%)	.092
* tropicalis*	15 (5.6%)	0	0	12 (8.5)	0	0	1 (2.3%)	2 (5.3%)	.4
* dubliniensis*	3 (1.1%)	1 (2.8%)	0	1	0	0	0	1 (2.6%)	.3
* metapsilosis*	4 (1.5%)	0	0	2 (1.4%)	0	0	0	2 (5.3%)	.4
* orthopsilosis*	2 (0.7%)	0	0	2 (1.4%)	0	0	0	0	>.9
* krusei*	2 (0.7%)	1 (2.8%)	0	1 (0.7%)	0	0	0	0	.4
* kefyr*	1 (0.4%)	1 (2.8%)	0	0	0	0	0	0	.2
* lusitaniae*	1 (0.4%)	0	0	1 (0.7%)	0	0	0	0	>.9
Antifungal susceptibility									
Fluconazole susceptible	20 (8.6%)	4 (11.1%)	0 (NA)	11 (9.2%)	0 (0.0%)	0 (0.0%)	5 (13.5%)	0 (0.0%)	.230
Voriconazole susceptible	10 (4.9%)	3 (8.6%)	0 (NA)	4 (3.9%)	0 (0.0%)	0 (NA)	1 (3.3%)	2 (5.4%)	.703
Posaconazole susceptible	5 (8.5%)	2 (100.0%)	0 (NA)	0 (0.0%)	0 (0.0%)	0 (NA)	0 (0.0%)	3 (13.0%)	.002
Amphotericin B susceptible	4 (1.8%)	0 (0.0%)	0 (NA)	3 (2.7%)	0 (0.0%)	0 (0.0%)	0 (0.0%)	1 (2.8%)	>.999
Echinocandins susceptible	29 (14.4%)	13 (36.1%)	0 (NA)	12 (10.8%)	0 (0.0%)	0 (NA)	4 (14.8%)	0 (0.0%)	<.001
5FC susceptible	5 (5.2%)	0 (NA)	0 (NA)	4 (7.8%)	0 (NA)	0 (NA)	0 (0.0%)	1 (2.7%)	.611
Itraconazole susceptible	1 (11.1%)	0 (NA)	0 (NA)	0 (0.0%)	0 (NA)	1 (50.0%)	0 (0.0%)	0 (NA)	.444
Surgical treatment									
DAIR	95 (36.0%)	12 (34.3%)	0	51 (36.7%)	0	1 (33.3%)	17 (39.5%)	14 (36.8%)	.8
2-stage exchange	78 (29.5%)	8 (22.9%)	3 (60.0%)	32 (23.0%)	1 (100%)	2 (66.7%)	16 (37.2%)	16 (42.1%)	.016
1-stage exchange	76 (28.8%)	12 (34.3%)	2 (40%	50 (36.0%)	0	0	6 (14.0)	6 (15.8%)	.019
Girdlestone	7 (9.3%)	1 (2.9%)	0	2 (1.4%)	0	0	4 (9.3%)	0	.14
Prosthesis removal	4 (1.5%)	0 (5.7%)	0	2 (1.4%)	0	0	0	0	.3
Amputation	2 (0.8%)	0	0	1 (0.7%)	0	0	0	1 (2.6%)	.6
Antifungal treatment									
Azoles	204 (85.4%)	8 (66.7%)	5 (100%)	120 (85.1%)	1	2 (66.6%)	32 (76.2%)	36 (100%)	.011
Echinocandins	83 (34.7%)	3 (25.0%)	0	59 (41.8%)	0	0	11 (26.1%)	10 (27.8)	.13
Amphotericin B	19 (7.9%)	0	0	10 (7.1%)	0	0	8 (19.0%)	1 (2.8%)	.2
Antifungal treatment duration. median (IQR), days	92.0 (53.0–182.0)	30.0 (13.0–57.0)	NA (NA–NA)	99.0 (70.0–185.0)	39.0 (39.0–39.0)	166.5 (113.0–220.0)	67.0 (50.0–140.0)	92.0 (46.0–181.0)	<.001
Outcome									
Cure	154	25 (69.4	4 (80)	86 (60.6)	1	1 (33.3%)	17 (39.5)	20 (52.6)	.063
Failure	113	10 (27.8	1 (20)	56 (39.4)	0	2 (66.7)	26 (60.5)	18 (47.8)	.037
Recurrence	52	2 (5.6	1 (20%)	27 (19.0)	0	1	12 (27.9	9 (23.7)	.14
Suppressive therapy	18	0 (0)	0	10 (7.0)	0	0	3 (7)	5 (13.2)	.4
Candida recurrence	21	1 (2.8)	0	11 (7.7)	0	0	5 (11.6)	4 (10.5)	.7
Candida other recurrence	14	1 (2.8)	0	9 (6.3)	0	0	4 (9.3)	0 (0)	.5
Nondocumented recurrence	18	0	1 (20%)	8 (5.6)	0	1 (33.3)	3 (7)	5 (13.2)	.058
Infective death	34	7 (19.4%)	0	16 (11.3)	0	1 (33.3)	7 (16.3)	3 (7.9)	.4
Other cause of death	9	1 (2.8)	0	3 (2.1)	0	0	4 (9.3)	1 (2.6)	.3
Recurrence delay	100 (47–375)	329 (175–484)	NA	91 (34–348)	NA	38	85 (58–182)	215 (91–396)	.2

Abbreviations: DAIR, debridement, antibiotics, and implant retention; IQR, interquartile range; NA, not available.

Immunosuppression showed pronounced heterogeneity (*P* = .001), ranging from only 2.8% in Austria to 28.9% in the United Kingdom. Conversely, comorbidity burden, assessed by the Charlson Comorbidity Index, did not differ between countries (*P* = .40).

History of previous PJI was frequent overall (75.7%) but varied substantially (*P* < .001), from 20.0% in Brazil to 85.9% in France or 84.2% in the United Kingdom. A previous *Candida* infection also demonstrated strong geographical variation (*P* < .001), being particularly common in Austria (30.4%), but rare in France (1.6%), and absent in several countries.

**Figure 1.**
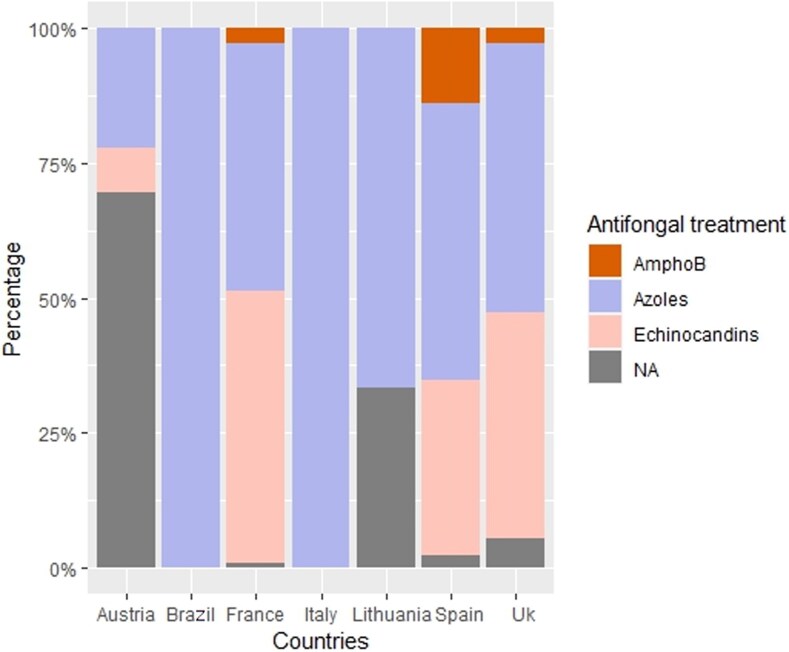
Distribution of Candida species ccording to countries during PJI due to Candida.

The anatomical distribution of infections was similar across countries, with no significant differences. Likewise, the distribution of *Candida* species remained broadly consistent (Figure 1), with *C albicans* (55.6%) and *C parapsilosis* (29.5%) predominating, and no statistically significant intercountry variation.

Across all settings, *C albicans* remained the predominant species, ranging from 41.6% in Austria to 100% in Italy and Lithuania. *C parapsilosis* showed numerically higher proportions in Austria (44%), Spain (39.5%), and the United Kingdom (23.7%), while being rare or absent elsewhere; however, these differences did not reach statistical significance, largely due to sparse data in several strata. *C glabrata* was observed mainly in France and the United Kingdom, and *C tropicalis* remained uncommon across all countries (≤8.5% in France), with no detectable geographical variation.

These findings were further supported by a multinomial logistic regression model evaluating the association between country and species distribution. The adjusted analysis similarly identified no statistically significant country-level effect. Although numerical disparities persisted—particularly the higher frequency of *C parapsilosis* in Austria, Spain, and the United Kingdom —the model did not demonstrate any independent association between geographical location and the likelihood of isolating a specific *Candida* species. This lack of significance is likely explained by the predominance of *C albicans* across all regions and the limited number of isolates in several countries.

Marked differences were observed in surgical management. Two-stage exchange was significantly more frequent in Brazil (60.0%) and the United Kingdom (42.1%) (*P* = .016), whereas 1-stage exchange was more commonly performed in Austria (34.3%) and France (36.0%) (*P* = .019). The proportion of debridement, antibiotics and implant retention (DAIR) procedures did not differ significantly across countries, with around one-third of all patients having this surgical option.

**Figure 2.**
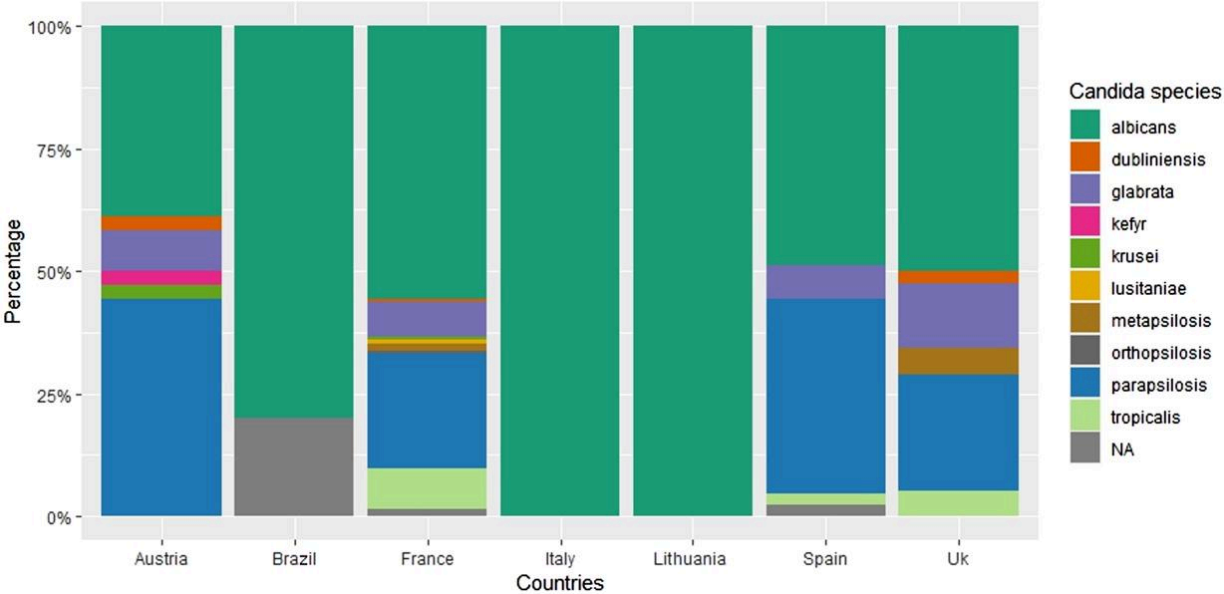
Antifungal treatment according to countries during PJI due to Candida.

Antifungal prescriptions also varied (Figure 2), with azole use showing the most significant disparity (*P* = .011), ranging from 66.7% in Austria and Lithuania to 100% in Brazil and the United Kingdom. Echinocandin and amphotericin B use did not differ statistically overall, but echinocandins were more prescribed in France (42%) whereas amphotericin B was more prescribed in Spain (19%). Overall, combination antifungal therapy was administered in 11.9% of cases (32/269).

Outcomes displayed borderline but noteworthy variability between countries. Cure rates ranged from 39.5% in Spain to 80.0% in Brazil (*P* = .063), while failure occurred in 42.2% of patients overall. Recurrence rates differed significantly (*P* = .037), with the highest proportion in Spain (27.9%) and the lowest in Brazil. Infective mortality (12.7%) and time to recurrence (median 100 days [47–375]) showed no significant geographical variation.

## DISCUSSION

In this large multicenter international cohort of *Candida* PJI, we identified substantial epidemiological heterogeneity between participating countries, yet striking microbiological homogeneity. Demographic and clinical characteristics, including age distribution, sex ratio, immunosuppression status, and history of prior PJI, varied widely across settings. For example, Austrian patients were significantly older, less frequently male, and rarely immunosuppressed, whereas patients from the United Kingdom had the highest prevalence of immunosuppression and one of the highest rates of previous PJI. Brazil contributed a markedly younger and predominantly male population with the lowest rate of prior PJI. These disparities likely reflect differences in referral patterns, local surgical practice, population structure, and indications for prosthetic joint implantation.

Despite these pronounced epidemiological differences, the microbiological landscape of *Candida* PJI remained remarkably consistent across countries. Neither univariate analyses nor adjusted multinomial regression identified any significant geographic variation in species distribution. *C albicans* predominated in every country, and although *C parapsilosis* was numerically more frequent in Austria, Spain, and the United Kingdom, these differences did not achieve statistical significance. Similarly, *C glabrata* and *Candida tropicalis* remained infrequent and evenly distributed, with no evidence of country-specific clustering. This was likely influenced by very small sample sizes in several contributing countries (eg, Brazil, Italy, Lithuania). The global absence of intercountry variation contrasts sharply with the well-described geographic differences observed in candidemia, where the rise of non-*albicans* species, including fluconazole-resistant *C parapsilosis* and *C glabrata,* is heterogeneously distributed across Europe and globally. Several explanations may account for this discrepancy. *Candida* PJI typically results from chronic low-inoculum contamination associated with biofilm formation and perioperative acquisition, rather than the acute bloodstream dissemination that characterizes candidemia. These infections may therefore reflect more stable, locally embedded ecological niches with less influence from antifungal selective pressures. Moreover, the low incidence of *Candida* PJI likely limits the emergence and transmission of clonal resistant lineages that are well documented in candidemia.

A recent review suggests that epidemiological patterns of fungal PJIs may vary between regions, particularly regarding the relative prevalence of *C albicans* versus non-*albicans Candida* species [4]. However, these differences should be interpreted cautiously, as most available data arise from heterogeneous retrospective series with variable reporting standards [3, 4, 8]. Similar variability is seen in management practices, including the use of antifungal susceptibility testing, the duration of therapy, and the choice between 1- and 2-stage revision, reflecting differences in local expertise and resource availability rather than clear evidence-based divergence. Overall, although regional trends can be observed, current data remain limited and do not yet allow firm conclusions about systematic epidemiological or therapeutic disparities across countries [4].

On the contrary, across the 3 large pan-European European Confederation of Medical Mycology studies conducted over 25 years, marked geographic heterogeneity emerged in the epidemiology of invasive *Candida* infections [9]. While *C albicans* remained predominant overall, its proportion declined nonuniformly across Europe, with a substantially higher burden of non-*albicans* species in northern countries. *C glabrata* was particularly common, mirroring higher fluconazole resistance rates in regions such as Belgium, the Czech Republic, Italy, Sweden, Turkey, and the United Kingdom. In contrast, *C parapsilosis* was more frequently isolated in southern Europe, where worrisome clusters of fluconazole-resistant strains were reported, especially in Greece, Italy, and Turkey. The extensive use of azoles in our cohort could suggest a presumed low prevalence of fluconazole resistance in the participating countries. Nevertheless, the high rates of treatment failure question this assumption and may indicate unrecognized resistance patterns or suboptimal antifungal decision-making, underscoring the need for improved stewardship strategies. The emergence of *Candida auris*, detected only in the most recent study, also displayed a patchy distribution, suggesting differing levels of surveillance capacity and diagnostic access across countries. These variations extended beyond species distribution to antifungal susceptibility patterns and guideline adherence, influenced by heterogeneous access to antifungal drugs and mycology expertise. Together, these findings underscore that invasive candidiasis in Europe does not represent a uniform epidemiological landscape but rather a mosaic shaped by regional practice patterns, healthcare structures, and antifungal availability [9].

These findings, together with the very low proportion of immunocompromised hosts, indicate that the epidemiology and clinical presentation of *Candida* PJI differ fundamentally from those of *Candida* bloodstream infections, which predominantly occur in immunocompromised patients and show substantial geographic variability. In contrast, *Candida* PJIs involve largely the same spectrum of species across settings and arise primarily in the context of prior surgery rather than underlying immunosuppression [2, 10]. *Candida* PJI should be regarded as a focal infection rather than a systemic disease.

Although surgical and therapeutic practices differed significantly between countries, most notably the more frequent use of 2-stage exchange in Brazil and the United Kingdom, and the wider use of azoles in some regions, these variations did not appear to influence species distribution. Indeed, each country used a limited choice of antifungals. Fluconazole was used in all countries but micafungin only in Spain and the United Kingdom. Amphotericin B, which is a very widely available antifungal worldwide, was only used in 3 countries. Anidulafungin was only used in Spain.

Outcome measures also showed limited geographical disparity, with only recurrence rates differing significantly and no detectable difference in mortality or time to recurrence. These findings suggest that, unlike candidemia, the management and prognosis of *Candida* PJI may be less driven by underlying microbiological variation and more by patient factors, surgical strategy, and healthcare system organization [1, 8, 11, 12].

Taken together, our results indicate that *Candida* PJI, despite occurring across diverse health systems and populations, displays a prominent microbiological uniformity that stands in contrast to the evolving and region-dependent epidemiology of invasive candidiasis. This observation has important clinical implications: in contrast to candidemia, empirical antifungal choices for *Candida* PJI may not require substantial regional adaptation, although local susceptibility patterns should always be considered [2, 10]. Future studies incorporating antifungal resistance profiling and genomic epidemiology will be essential to determine whether subtle geographic differences exist that could not be detected in this cohort and to assess the potential emergence of resistant biofilm-associated strains.

This study has limitations. Its retrospective design and voluntary participation may have introduced selection bias and incomplete data capture. Although representing one of the largest international cohorts of Candida PJI, case numbers remained limited in several countries, reducing statistical power. In addition, antifungal treatment data were unavailable for a substantial proportion of cases, which may have affected the interpretation of therapeutic practices and outcomes.

## CONCLUSION

In this large international cohort of *Candida* PJI, substantial epidemiological heterogeneity contrasted with a remarkably uniform microbiological profile. Unlike candidemia, where species distribution and antifungal resistance vary widely across regions, *Candida* PJI demonstrated no significant intercountry differences in species epidemiology. *C albicans* remained consistently predominant, and no country-level effect on species distribution was detected after adjustment. These findings suggest that the microbiology of *Candida* PJI may be shaped less by regional antifungal pressures and more by intrinsic features of biofilm-associated perioperative infection. Despite notable variation in surgical management and antifungal prescribing practices, clinical outcomes were broadly comparable across countries. Altogether, our results support the need for harmonized international guidelines for the diagnosis and management of *Candida* PJI, while highlighting the importance of continued surveillance to detect potential shifts in species ecology or emerging antifungal resistance.
